# Drug repurposing screen identifies lestaurtinib amplifies the ability of the poly (ADP-ribose) polymerase 1 inhibitor AG14361 to kill breast cancer associated gene-1 mutant and wild type breast cancer cells

**DOI:** 10.1186/bcr3682

**Published:** 2014-06-24

**Authors:** Guelaguetza Vazquez-Ortiz, Cristine Chisholm, Xiaoling Xu, Tyler J Lahusen, Cuiling Li, Srilatha Sakamuru, Ruili Huang, Craig J Thomas, Menghang Xia, Chuxia Deng

**Affiliations:** 1 Genetics of Development and Disease Branch, National Institute of Diabetes, Digestive and Kidney Diseases, National Institutes of Health, 9000 Rockville Pike, Bethesda, MD 20892, USA; 2 NIH Chemical Genomics Center, National Center for Advancing Translational Sciences, National Institutes of Health, 9800 Medical Center Drive, Rockville, MD 20850, USA

## Abstract

**Introduction:**

Breast cancer is a devastating disease that results in approximately 40,000 deaths each year in the USA. Current drug screening and chemopreventatitive methods are suboptimal, due in part to the poor specificity of compounds for cancer cells. Poly (ADP-ribose) polymerase 1 (PARP1) inhibitor (PARPi)-mediated therapy is a promising approach for familial breast cancers caused by mutations of breast cancer-associated gene-1 and -2 (BRCA1/2), yet drug resistance frequently occurs during the treatment. Moreover, PARPis exhibit very little effect on cancers that are proficient for DNA repair and clinical efficacy for PARPis as single-agent therapies has yet to be illustrated.

**Methods:**

Using a quantitative high-throughput screening approach, we screened a library containing 2,816 drugs, most of which are approved for human or animal use by the Food and Drug Administration (FDA) or other countries, to identify compounds that sensitize breast cancer cells to PARPi. After initial screening, we performed further cellular and molecular analysis on lestaurtinib, which is an orally bioavailable multikinase inhibitor and has been used in clinical trials for myeloproliferative disorders and acute myelogenous leukemia.

**Results:**

Our study indicated that lestaurtinib is highly potent against breast cancers as a mono-treatment agent. It also strongly enhanced the activity of the potent PARPi AG14361 on breast cancer cell growth both *in vitro* and *in vivo* conditions. The inhibition of cancer growth is measured by increased apoptosis and reduced cell proliferation. Consistent with this, the treatment results in activation of caspase 3/7, and accumulation of cells in the G2 phase of the cell cycle, irrespective of their BRCA1 status. Finally, we demonstrated that AG14361 inhibits NF-κB signaling, which is further enhanced by lestaurtinib treatment.

**Conclusions:**

Lestaurtinib amplifies the ability of the PARP1 inhibitor AG14361 to kill BRCA1 mutant and wild-type breast cancer cells, at least in part, by inhibiting NF-κB signaling. Each of these drugs has been approved for clinical trials for several different cancers, thus, their combination treatment should be applicable for a breast cancer trial in the future.

## Introduction

Breast cancer is one of the most prevalent cancers in women worldwide and it is estimated that a million women will develop this disorder each year. About 8% of breast cancer cases are inheritable, associated with mutations of highly penetrant breast cancer susceptibility genes, such as breast cancer-associated gene-1 and -2 (*BRCA1*/2) and other tumor suppressor genes [1-7]. In addition, it has been estimated that BRCA1 mutation carriers have a 50 to 80% risk of developing breast cancer before the age of 70 years [8-11].

Both BRCA1 and BRCA2 play essential roles in many biological processes [12-17]. A common feature of BRCA1/2-associated tumorigenesis is massive genetic instability, primarily due to the fact that cells lacking BRCA1 or BRCA2 have impaired ability to undergo homologous recombination (HR) [15,16,18,19], therefore these cells cannot effectively repair HR-mediated DNA damage, including DNA double-strand breaks (DSBs). The genetic instability often leads to altered expression of many genes and signaling pathways making it difficult to inhibit tumorigenesis and progression by targeting a single molecular target. Recently, significant work in the area of synthetic lethality has led to new approaches for the treatment of BRCA1/2-deficient cancers using high efficacy poly(ADP-ribose) polymerase 1 (PARP1) inhibitors (PARPi) with high efficiency [20-24].

The PARP family plays important roles in DNA damage repair. For example, PARP1 is involved in the repair of DNA single-stranded breaks (SSBs) [25-27]. Inhibition of PARP1 activity could also result in DSB formation when unrepaired SSBs meet the replication fork, causing its collapse. Because BRCA1/2 mutant cells are defective in repairing DSBs, PARPi inhibition may result in accumulation of DSBs in these cells and eventually lead to apoptosis. This may account for the molecular basis of why BRCA1- and BRCA2-deficient cells are extremely sensitive to PARPi [20-22]. However, it was shown that several cell lines derived from mouse BRCA1 mutant mammary cancers [28] and a human pancreatic BRCA2 mutant cancer cell line [29] exhibited resistance to PARPi. While the exact cause for the resistance was unclear, it was hypothesized that some specific alterations/mutations might block the sensitivity of these cancer cells to PARPi [30]. It was subsequently demonstrated that resistance to PARPi could occur through multiple mechanisms (reviewed in [31]), such as impaired expression of 53BP1 [32,33], restoration of BRCA function [34], and induction of P-glycoprotein expression [35,36]. To overcome the resistance, combinational therapies using multiple chemotherapeutic agents have been used to enhance the ability to kill BRCA1/2-related cancers [35,36].

In theory, all clinically used drugs have effects on biological systems other than those for which they were designed; therefore, drug repurposing consists of developing new applications for existing drugs. There has been an increase in the interest of drug repurposing due to the high cost of drug development and time involved in bringing new drugs to the market [16,37]. It has been estimated that it costs approximately more than USD 800 million to develop a new drug *de novo* and the time estimated to develop a new drug that complies with the regulatory requirements for safety, efficacy and quality goes in the order of 10 to 17 years [38].

In this study, a drug repurposing approach using the National Institutes of Health Chemical Genomics Center (NCGC) Pharmaceutical Collection (NPC) [39], a library containing drugs approved for clinical use or that have been in clinical trials, was used to identify drugs that amplify the ability of AG14361, a potent PARP1 inhibitor [21], to inhibit the growth of both human and mouse breast cancer cells, irrespective of their BRCA1 status.

## Methods

### Cell lines and viral vectors

Our initial study for human cell lines was performed in three isogenic models derived from the primary cell lines: 92 J, MDA-MB-231 (American Type Culture Collection, ATCC) and T47D (ATCC) and their BRCA1 mutant sublines 92 J-sh-BRCA1, MDA-MB-231-sh-BRCA1 and T47D-sh-BRCA1 respectively. The 92 J cell line, which is derived from a xenograft tumor of MDA-MB-231, forms mammary tumors much faster than the parent MDA-MB-231 cells when implanted into nude mice.

BRCA1 short hairpin RNA (shRNA) constructs in the pLKO.1-based vector were obtained from Open Biosystems (GE Healthcare, Little Chalfont, UK). A control lentiviral shRNA vector, packaging vector pCMV-dR8.2, and envelope vector VSV-G was obtained from Addgene (Cambridge, MA, USA). The BRCA1 shRNA construct (TRCN0000039837) was used to produce lentiviral particles for generation of stable BRCA1 knockdown cells. Lentivirus was produced in 293 T cells and the media collected for later transduction of target cells. Cells were transduced with lentiviral supernatant and then selected with 2 μg/ml puromycin to generate cells with stable knockdown of BRCA1. The viral supernatant was used to infect 92 J, MDA-MB-231 and T47D cells

Mouse BRCA1 mutant cell line 69 derived from mammary tumor of *Brca1*^*Co*/*Co*^*; MMTV-Cre;p53*^+/−^ mice containing a targeted deletion of full-length BRCA1 [40] and BRCA1 wild-type (wt) cell line Ras, derived from mammary tumor MMTV-Ras mice [41].

### Growth assays

For the growth curve assay, 5 × 10^4^ cells were plated per well of a six-well plate and medium was changed every 24 hr. The plate was incubated at 37°C in 5% CO_2_ and every 12 hr cells were detached by trypsinization and counted with a Z1 Coulter counter (Beckman Coulter, Brea, CA, USA). Plating for each time point was done in triplicate for each 92 J isogenic pair. In order to eliminate artifacts that could be produced by the cell line, we validated the other two pairs of human isogenic cell lines following the same protocol.

### NCGC Pharmaceutical Collection (NPC) and quantitative high throughput screening

The NPC drug library consists of 2,816 small molecule compounds at the time of this screening [39]. Fifty-two percent of the compounds are drugs approved for human or animal use by the United States Food and Drug Administration (FDA), 22% are drugs approved in Europe, Canada or Japan, and the remaining 25% are drugs approved in other countries or compounds that have been tested in clinical trials.

For the initial screening, the library was prepared as 15 inter-plate titrations, which were serially diluted 1:2.236 in dimethyl sulfoxide (DMSO, (Thermo Fisher Scientific, Waltham, MA, USA) in 384-well plates. The stock concentrations of the test compounds ranged from 10 mM to 0.13 μM. Transfer of the diluted compounds from 384-well plates to 1,536-well plates was performed using an Evolution P3 system (Perkin Elmer, Wellesley, MA, USA). Each treatment plate included concurrent DMSO and positive control wells and concentration-response titrations of controls, all occupying columns 1 to 4. Cell viability was measured using a luciferase-coupled ATP quantization assay of metabolically active cells (ATPliteTM 1step Luminescence Assay System, Perkin Elmer). Cells were dispensed at 2,000 cells/5 μL/well in 1,536-well white, solid-bottom assay plates using a flying reagent dispenser (FRD). The assay plates were incubated at 37°C for 5 hr to allow for cell attachment, followed by addition of 5 μl of compounds via pin tool. After compound addition, plates were incubated for 48 hr at 37°C. At the end of the incubation period, 5 μL of ATPlite reagent was added, plates were incubated at room temperature for 20 to 30 min, and luminescence intensity was determined using a ViewLux plate reader (Perkin Elmer).

### Data analysis

Analysis of compound concentration-response data was performed as previously described [36]. Briefly, raw plate reads for each titration point were first normalized relative to the positive control compound (-100%) and DMSO-only wells (0%) as follows: % activity = ((V_compound_ – V_DMSO_)/(V_pos_ – V_DMSO_)) × 100, where V_compound_ denotes the compound well value, V_pos_ denotes the median value of the positive control wells, and V_DMSO_ denotes the median values of the DMSO-only wells, and then corrected by applying a NCGC in-house pattern correction algorithm using compound-free control plates (DMSO-only plates) at the beginning and end of the compound plate stack. Concentration-response titration points for each compound were fitted to a four-parameter equation yielding concentrations of half-maximal activity (AC_50_) and maximal response (efficacy) values. Compounds were designated as class 1 to 4 according to the type of concentration-response curve observed [42,43]. Curve classes are heuristic measures of data confidence, classifying concentration-responses on the basis of efficacy, the number of data points observed above background activity, and the quality of fit. Compounds with class 1.1, 2.1, 1.2 or 2.2 (>50% efficacy) curves are considered active. Compounds with class 4 curves are considered inactive and compounds with all other curves classes are considered inconclusive. Compounds that were selectively active (showed a potency difference of >3-fold) in one cell line or with or without the combination compound were selected for confirmation and follow-up studies.

### Determination of synergistic effect and additive effect

The theoretical addictive effect of compounds with AG14361 was based on the fractional inhibition of these compounds when used separately. If the 50% inhibition concentration (IC_50_) of each drug is administered together, by the union of two events, the predictive addictive killing is calculated as E_total_ = E_1_ + E_2_ – E_1_ × E_2_ (where E_1_ is IC_50_ of drug 1 and E_2_ is IC_50_ of drug 2), which is 75%. This classifies a drug synergistic if, when treated with the 50% inhibition dose of each drug, the synergistic killing effect should be significantly greater than 75%.

### Cell proliferation assays for validation of synergistic effect

In order to validate the synergistic effect of the selected drugs *in vitro* we performed cell viability assay using a luciferase-coupled ATP quantization assay of metabolically active cells (ATPliteTM 1step Luminescence Assay System, Perkin Elmer) in a 96-well plate and 3-(4,5-dimethylthiazol-2-yl)-2,5-diphenyl tetrazolium bromide (MTT). For MTT, 1 to 2 × 10^4^ cells were plated per one well of a 24-well plate. Target drugs at various concentrations were dissolved in DMSO and then added to the cells in 10% fetal bovine serum-containing Dulbecco’s modified Eagle’s medium (DMEM), IC_50_ concentration of AG14361 were also added to each well. The final DMSO concentration was kept at 0.1% after the addition to medium. After 48 hr medium was removed and 0.3 ml of 0.1% MTT in phosphate-buffered saline (PBS) was added in each well. After incubation for 30 min in a 37°C CO_2_ incubator, MTT solution was removed and 0.8 ml of 2-propanol was added. After shaking for 30 min, OD560 was measured using a plate reader. Plating for each time point was done in triplicate.

### Histological and immunohistochemical analysis of tumor samples

For immunohistochemistry procedures, the tumors were fixed in phosphate-buffered formalin, embedded in paraffin, cut in 4-μm thickness, and stained. Immunohistochemical analysis of proliferating cell nuclear antigen (PCNA) was performed using a labeled streptavidin-biotin technique described previously. Anti-PCNA monoclonal antibody PC 10 (Dako, Carpenteria, CA, USA), which reacts exclusively with nuclei, was used at a dilution of 1:200. The number of PCNA-positive cells was counted in five high-power fields (0.135 mm^2^ fields at × 200 magnification) selected at random, and the PCNA labeling index for each field was calculated as the percent of PCNA-positive cells (relative to the total). Apoptosis in tumor cells was detected using the terminal deoxynucleotidyl transferase-mediated dUTP-biotin nick end-labeling (TUNEL) assay, as described previously. In the same manner as PCNA, five fields (0.135 mm^2^ fields at × 200 magnification) were selected at random, and the apoptotic index of each field was calculated as the percentage of TUNEL-positive cells.

### RT-PCR and real-time PCR

Total RNA from cells or tissues were extracted with RNA STAT-60™ following the manufacturer’s protocol (Tel-Test, Inc., Gainesville, FL, USA), and cDNA was generated by Cells-to-cDNA™II (Ambion, Inc., Austin, TX, USA). Quantitative RT-PCR was performed using a SYBR green PCR Master Mix (Applied Biosystems, Carlsbad, CA, USA) and the 7500 Real-Time PCR system (Applied Biosystems). Primers used are listed below: BRCA1F 5′-ctgatgtgctttgttctgga-3′ BRCA1R: 5′-ggctatcctctaagagtgaca-3′, β-actinF: 5′gatggagttgaagtgagtttcgtg-3′, β-actinR 5-gcgggaaatcgtgcgtgcgtgacatt-3′, IL8F: 5′-aatctggcaaccctagtctgcta-3′, IL8R: 5′-aaaccaaggcacagtggaaca-3′, p50F: 5′-cagctcttctcaaagcagca-3′, p50R: 5′tccaggtcatagagaggctca-3′, MMP9F: 5′-cctgtgtgttcccgttcatct-3′, MMP9R: 5′-cgctggaatgatctaagccca-3′, COX2F: 5′-gaagtggggtttaggatcatc-3′, COX2R: 5′-cctttcactttcggataacca-3′, p65F: 5′-gcaggctcctgtgcgtgtct-3′, p65R: 5′-ggtgctcagggatgacgtaaag-3′, IL6F: 5′-cactgggcacagaacttatgttg-3′, IL6R: 5′-aaaataattaaaatagtgtcctaacgctcat-3′.

### Luciferase reporter assay

The reporter plasmid, pNF-κB-luc, containing the κB-enhancer consensus sequences ((TGGGGACTTTCCGC) × 5) and nuclear factor κB (NF-κB)-dependent firefly luciferase gene was purchased from Stratagene (La Jolla, CA, USA). 92 J, and MDA-MB-231 isogenic cells were transiently transfected with two plasmids (pNF-κB-luc plasmid and renilla) using the LipofectAMINE 2000 Plus regent. Cells were seeded in a 24-well plate the day prior to transfection to achieve 80 to 85% confluence on the following day. Twelve hours after transfection, cells were incubated for an additional 24 hr in medium containing IC_50_ of AG14361, and lestaurtinib as mono-treatment and in combination and harvested for luciferase reporter assays. NF-κB transcription activity assay was also performed in a HeLa cell line, which carries a stably integrated luciferase reporter (Signosis, Inc., Santa Clara, CA, USA) after treatment with IC_50_ of AG14361 and/or lestaurtinib for 24 hr. Luciferase activity was measured with a luminometer using the Luciferase Assay System (Signosis). Renilla activity was detected to normalize any variations.

### Caspase 3/7 activity

The 92 J isogenic cell lines were dispensed in culture medium at 2,000 cells/5 μl/well in 1536-well white/solid-bottom assay plates. The cells were incubated a minimum of 5 hr at 37°C. The compounds (23 nl/well) were added via the pin tool and then Resveratrol or AG14361 were added. The treated cells were incubated for 5 or 24 hr at 37°C, followed by the addition of the Caspase-Glo 3/7 (Promega, Madison, WI, USA) reagent at 5 μl/well. After 30 min incubation at room temperature, the luminescence intensity of the assay plates was measured using a ViewLux Plate Reader (Perkin Elmer).

### Cell cycle analysis

After the 12, 24, 36 and 48 hr of drug treatment as mono-treament and combination, 92 J cells were trypsinized and washed twice in PBS (pH 7.4). Cells were fixed in 2 mL 70% ethanol (stored at −20°C), vigorously vortexed and incubated at 4°C for 4 hr. The cells were then washed with ice-cold PBS and resuspended in 200 μl PBS. Subsequently, cell suspension was incubated with 20 μl DNase-free RNase (10 mg/mL) and 1 ml of DNA intercalating dye PI (50 μg/ml, Triton-X 100 1.0%) at 4°C for 30 min. Cell cycle phase analysis was performed by flow cytometry using Epics-XL II FACS Caliber flow cytometer (Beckman Coulter, Brea, CA, USA), and data were analyzed by Multicycle AV software (Phoenix Flow Systems, San Diego, CA, USA).

### Tumor formation assay in nude mice

Ninety-two J-shBRCA1 and 92 J-PLK cells after trypsinization were resuspended in PBS, the cells were injected into the fourth mammary fat pad on both sides of female nude mice at 1 × 10^6^ cells/100 μl/spot. There were four groups of mice per cell line injected defined by the drug they were treated by. The first group was injected with PBS, second group with lestaurnitib, the third with AG14361, and the fourth with the combination of lestaurnitib and AG14361. Each group was formed of seven mice, with a total of twenty-eight mice per studied cell line. The drug treatment started when tumors became palpable (about 14 days after cell implantation). The mice were injected with AG14361 (30 mg/kg) intraperitoneally five times per week and lestaurtinib (10 mg/kg) three times per week, respectively. The recipient mice were housed in pathogen-specific facility, kept in a 12 hr light and dark cycle, and fed with a regular diet. Mice were monitored for tumor formation, and were euthanized when the tumors were 3.5 cm, which required harvesting, or there were tumor ulcerations. Tumor size was measured every three days before day 15 and every four days thereafter with a caliper, and tumor volume was calculated by using the formula V = 2/3πr_x_r_y_r_z_ (r is radius and x, y, z refer to each axis, and π = 3.14). All animal experiments were approved by the Animal Care and Use Committee of National Institute of Diabetes, Digestive and Kidney Diseases (ACUC, NIDDK).

### Statistical analyses

All analyses were performed with the assistance of GraphPad Prism software version 4.0a (GraphPad Software, San Diego, CA, USA). A *P* value of less than 0.05 was considered statistically significant.

## Results

### Isogenic cell lines carrying acute knockdown of BRCA1 expression

**Figure 1:**
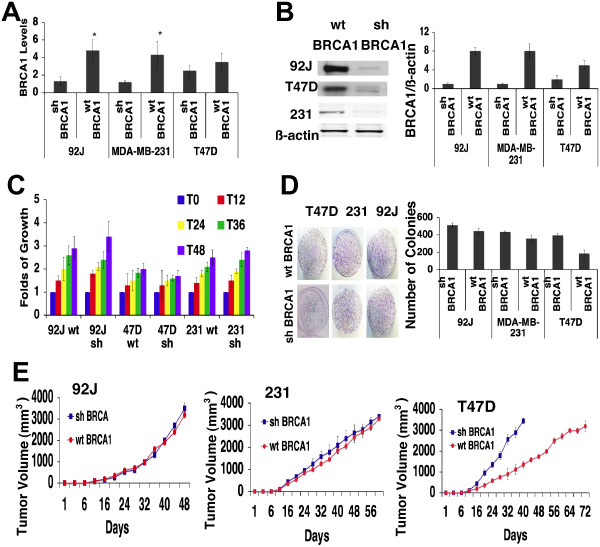
**Human isogenic cell lines proliferation *in vitro *and tumor growth in xenografts. (A)** Reduction in the levels of expression of BRCA1 in 92 J, MDA-MB-231 and T47D cells transduced with shBRCA1 lentivirus were determined by qRT-PCR and by Western blot analysis. **(B)** Western blot analysis showing BRCA1 levels. Immunoblot photographs of three independent experiments were also subjected to densitometry (right panel). **(C)** Fold change in cell growth rate in respect of the initial input of cells was measured in human isogenic cell lines. Data shown represent mean standard deviation (SD) from triplicate experiments. **(D)** Clonogenic assays performed on the three pair of isogenic cells. A total of 1,000 cells were seeded in 10 ml plates and grown for 21 days and counted at the end point. Data represent the mean SD from triplicate experiments. **(E)** 92 J, MDA-MB-231 and T47D isogenic cells were injected in the mammary fat pad of immunocompromised mice and the tumor volume was measured when they reached 3.5 mm^3^. Each experiment group consisted of five mice and each mouse had two tumors. The measurements on the graph represent the average of ten tumors per group. shBRCA1, sample with short hairpin RNA (shRNA) against breast cancer type 1 susceptibility (BRCA1).

In order to screen the NCGC Pharmaceutical Collection (NPC) Library, we generated three pairs of human isogenic cancer cell lines from T47D, MDA-MB-231, and 92 J, which were derived from an xenograft tumor in nude mice implanted with MDA-MB-231 cells infected them with lentivirus carrying an shBRCA1 or a control (PLK) vector. Downregulation of BRCA1 mRNA and protein were verified by qRT-PCR (Figure 1A), and Western blot (Figure 1B). We tested levels of proliferation by MTT (Figure 1C), clonogenic assays (Figure 1D), and tumor growth (Figure 1E) of these isogenic cell lines, and found that the 92 J pair (92 J-shBRCA1 and 92 J- wtBRCA1) and MDA-MB-231 pair (MDA-MB-231-shBRCA1 and MDA-MB-231-wtBRCA1) exhibited similar growth, irrespective of their BRCA1 status (Figure 1E). The growth of T47D-shBRCA1 was slower than that of T47D-wtBRCA1 (Figure 1E), therefore, this pair of cells was not suitable for drug screening in our further experiment. We have also tested two Brca1-deficient mouse cell lines, 780 and 69, and two Brca1 wild-type cell lines, NK and Ras, which were derived from mammary tumors of MMTV-Neu and MMTV-Ras, respectively [40,41]. The cell lines 69 and Ras showed similar proliferation rate (Additional file 1A), colony formation (Additional file 1B), and tumor growth in nude mice (Additional file 1C), therefore they were used for further studies.

### Screening for drugs that synergistically kill breast cancer cells in the presence of PARP1 inhibitor AG14361

To identify small molecule drugs that can synergize with PARPi for breast cancer cells, the 92 J isogenic pair of cell lines was used to screen the NPC library in the absence or presence of AG14361 at a concentration that killed 50% (IC_50_) of cells (92 J-wt-BRCA1, 17 μM, 92 J-sh-BRCA1, 25 μM, respectively). Compounds were prepared in 15 concentrations with 2.236-fold dilution titrations in DMSO, with final concentrations ranging from 0.11 pM to 92 μM. This identified a total of 32 compounds that showed both high potency as mono-treatment (<30 μM) and had a synergistic effect with AG14361 (Additional file 2: Table S1). Among these 32 compounds, 17 showed similar levels in killing both shBRCA1 and wtBRCA1 cells, six were more specific for killing shBRCA1 cells; and the remaining nine killed wtBRCA1 cells better.

Next, we focused on 17 compounds that showed similar levels in killing both shBRCA1 and wtBRCA1 cells. Confirmation studies were performed with 92 J cells on a new aliquot of these compounds taken from the original library. Strong synergistic activity was confirmed in 11 of the 17 compounds tested (The first 11 in the Table S1 in Additional file 2). The remaining six compounds, which showed relatively weak synergy with AG14361 in the initial screen (Table S1 in Additional file 2), exhibited even weaker synergy (see Additional file 3).

### Lestaurtinib and its combination with AG14361 on breast cancer cell lines growth *in vitro*

**Figure 2:**
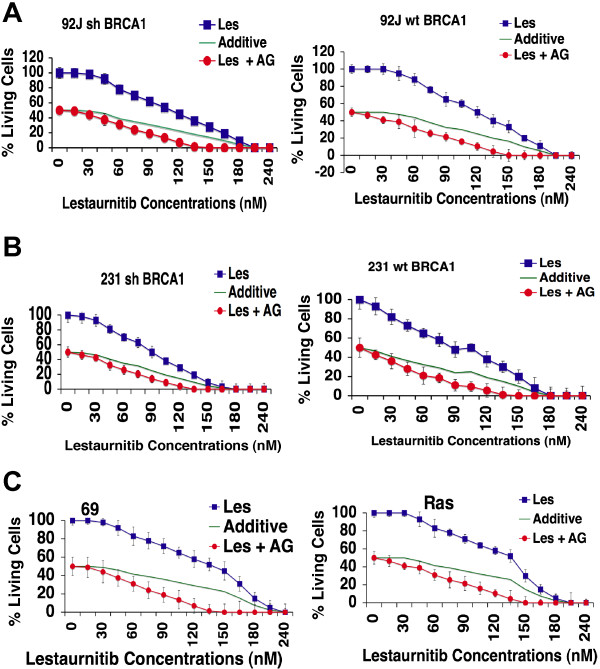
**Synergistic effect of lestaurtinib in combination with AG14361 *in vitro*.** Synergistic effect of lestaurtinib in the presence of IC_50_ AG14361 on 92 J isogenic cell lines **(A)**, MDA-MB-231 isogenic cell lines **(B)**, and 69 and Ras cell lines **(C)** revealed by ATP release assay. Data shown represent mean ± standard deviation (SD) from triplicate experiments. IC_50_, 50% inhibition dose.

Among 11 compounds that showed similar levels in killing both BRCA1 mutant and wild-type cells in the presence of AG13461, we decided to focus on lestaurtinib (CEP-701) first. Lestaurtinib is a tyrosine kinase inhibitor and has been used in several clinical trials for myeloproliferative disorders and acute myelogenous leukemia [44-46]. In order to independently verify the activity and synergistic effect of lestaurtinib with AG14361, we performed ATP release assay in a 96-well format with lestaurtinib that was obtained from a different vendor. Besides the 92 J pair of cell lines, an additional human isogenic pair of breast cancer cell lines (MDA-MB-231-wtBRCA1 and MDA-MB-231-shBRCA1), and two mouse mammary cell lines, Ras and 69, were also used in the assay. The data confirmed the synergistic cytotoxic effect of lestaurtinib with AG14361 in all three pairs of cell lines (Figure 2). We also performed cell proliferation assays using MTT with a 24-well format, and obtained similar results (data not shown).

### Lestaurtinib and its combination with AG14361 on mouse breast cancer cell growth *in vivo*

**Figure 3:**
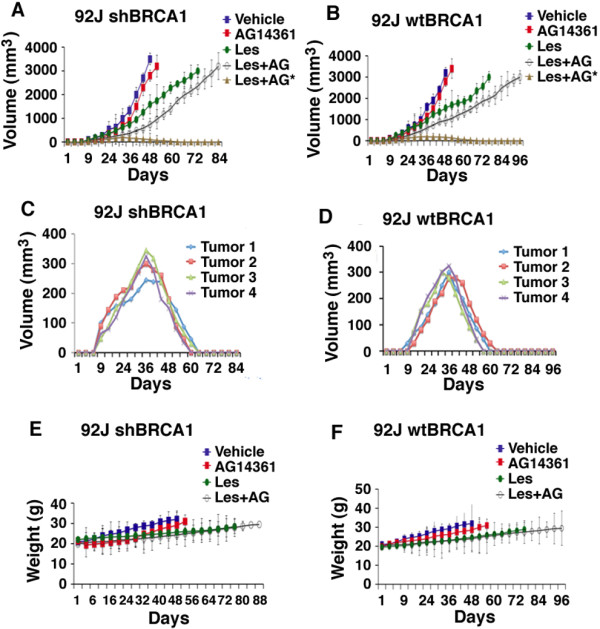
**Synergistic effect of lestaurtinib with AG14361 on inhibiting breast cancer tumors in allografts.** Tumor progression **(A-D)** and body weight of recipient mice **(E,F)** and all experimental mice. **(C,D)** show tumor progression in four mice with complete tumor regression upon the combined treatment of lestaurtinib and AG14361 (indicated as Les + AG* in panels A and B). The day of implantation was designated as day 0 and the drug or vehicle was administered intraperitoneally to nude mice that had developed 92 J-wtBRCA1 and 92 J-shBRCA1 xenograft tumors of 50- to 100-mm^3^ volume (about 14 days after cell implantation). shBRCA1, sample with short hairpin RNA (shRNA) against breast cancer type 1 susceptibility (BRCA1); wtBRCA1, sample with wild-type BRCA1 status.

**Figure 4:**
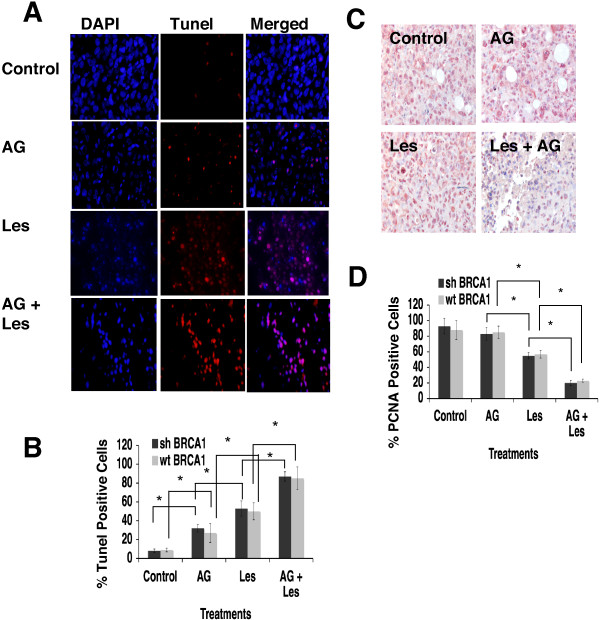
**Analysis of tumors treated with AG14361 and lestaurtinib as mono-treatment and combination therapy.** Images **(A)** and quantification **(B)** of TUNEL assay for xenograft tumors from 92 J-shBRCA1 and 92 J-wtBRCA1 cell lines. **(C, D)** Images **(C)** and quantification **(D)** of PCNA immunochemical staining for the same tumors used in **(A, B)**. Data show the mean of three different readings and are marked by columns with ± standard deviation (SD) error bars. PCNA, proliferating cell nuclear antigen; shBRCA1, sample with short hairpin RNA (shRNA) against breast cancer type 1 susceptibility (BRCA1); TUNEL, terminal deoxynucleotidyl transferase-mediated dUTP-biotin nick end-labeling; wtBRCA1, sample with wild-type BRCA1 status.

We compared the responses of mice bearing 92 J-shBRCA1 and 92 J-wtBRCA1 xenograft tumors to treatment with vehicle, AG14361 and lestaurtinib, alone or in combination. For tumors derived from both types of cells, mono-treatment of AG14361 slightly delayed tumor growth compared with the vehicle group, and lestaurtinib treatment significantly delayed tumor growth, that took tumors 30 more days, on average, to reach a similar size compared with the tumors in the vehicle-treated group (Figure 3A,B). Of note, the combination of AG14361 and lestaurtinib resulted in complete tumor regression in two 92 J-shBRCA1 mice and two 92 J-wtBRCA1 mice (Figure 3A,B). In these cases, the tumors initiated at about day 9 and reached about 300 mm^3^ at day 36, and then they gradually reduced volume and eventually disappeared (Figure 3C,D). The growth of the remaining 10 tumors treated with lestaurtinib/AG14361 was delayed about 20 days compared with tumors with lestaurtinib mono-treatment. During the treatment, the recipient mice gradually increased their body weight, which was proportional to the progression of their tumors (Figure 3E,F), suggesting that the treatment did not cause an adverse effect on animal health, as measured by weight loss. Thus, the combined lestaurtinib and AG14361 markedly delayed tumor growth in all treated mice and even caused complete regression in four out of fourteen tumors derived from 92 J-shBRCA1 and 92 J-wtBRCA1 cells, respectively. Because our data clearly indicate that these tumors initiated in the beginning and were gradually regressed later (Figure 3C,D) and such regression was not observed in the 42 tumors with vehicle, lestaurtinib or AG14361 mono-treatment derived from each cell line, we believe the regression is caused by the combined treatment of lestaurtinib and AG14361. This suggests that the combination of lestaurtinib and AG14361 could be useful in a therapeutic setting.Next, we examined cell proliferation and apoptosis in tumors of control and drug-treated mice. There was an increase in TUNEL-positive cells in tumors with an increasing order from no treatment, AG14361, lestaurtinib, to AG14361/lestaurtinib (Figure 4A,B). The apoptotic index of the combination therapy reached approximately 90%, which was significantly higher than those from mice with lestaurtinib (55%), and AG14361 (30%) mono-treatment, whereas no significant difference between shBRCA1 and wtBRCA1 tumors was detected (Figure 4B). Immunochemical staining using an antibody for proliferating cell nuclear antigen (PCNA) detected a reversed order of decreasing PCNA-positive cells from these tumors (Figure 4C,D). Thus, the inhibition of tumor growth of these treatments is correlated with increased apoptosis and decreased cell proliferation.

### Treatment results in caspase 3/7 activation and cell cycle abnormalities

**Figure 5:**
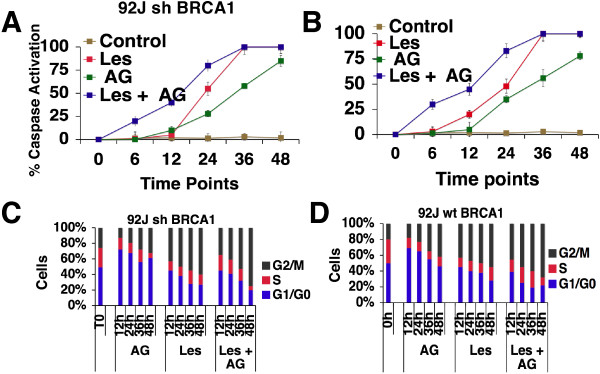
**Caspase 3/7 activity and cell cycle distribution of 92 J isogenic cell lines upon lestaurtinib and/or AG14361 treatment. (A, B)** Percentage of caspase 3/7 activity in 92 J isogenic pair of cells after they were incubated with IC_50_ of lestaurtinib and/or AG14361 in a time-dependent manner. **(C, D)** Distribution of 92 J isogenic pair of cells after they were treated with IC_50_ of AG14361 and/or lestaurtinib per 12, 24, 36 and 48 hr. There was no difference in the results between the two isogenic cell lines. IC_50_, 50% inhibition dose.

To understand the cause of the enhanced apoptosis, caspase 3/7 activity was measured in the 92 J pair of cells after treatment with AG14361 and/or lestaurtinib at their IC_50_ concentrations. Our data demonstrated that all the treatments activated caspase 3/7 in a time-dependent manner. As a mono-treatment, lestaurtinib was a more potent stimulator of caspase 3/7 activity than AG14361, and the combination of both drugs significantly enhanced this activity during the period of treatment of both cell lines, especially before 36 hr (Figure 5A,B).Next, we determined the cell cycle distribution of 92 J-shBRCA1 and 92 J-wtBRCA1 cells after different drug treatments at four time points using flow cytometry. The proportion of cells in the G1 phase was markedly increased upon treatment of AG14361 at 12 hr at the expense of S and G2 phases as compared to untreated cells (Figure 5C). From 12 hr to 48 hr, AG14361 treatment resulted in gradually decreased fraction of cells in the G1 phase and increased in the G2 phase, while a more dramatic reduction in S phase was observed at 48 hr. This data is consistent with the finding that AG14361 significantly reduced cell proliferation. Compared with untreated cells, lestaurtinib at 12 hr did not have an obvious effect on G1 phase, instead it caused expansion of G2 phase and reduction of S phase. From 12 hr to 48 hr treatment, lestaurtinib resulted in gradually decreased G1 phase and increased G2 phase, while it did not cause an obvious change in S phase (Figure 5C). Combined treatment of AG14361 and lestaurtinib caused further expansion of cells at G2, which is similar to, but more severe than the mono-treatment with lestaurtinib. Moreover, it also caused a marked decrease in the S phase population, which is similar to the mono-treatment of AG14361. Both cell lines showed similar responses (Figure 5D). Thus the treatment of both drugs recapitulated features of mono-treatment of these two drugs, consequently leading to increased apoptosis and inhibition of cell proliferation.

### AG14361 inhibits NF-κB signaling that is enhanced by lestaurtinib treatment

**Figure 6:**
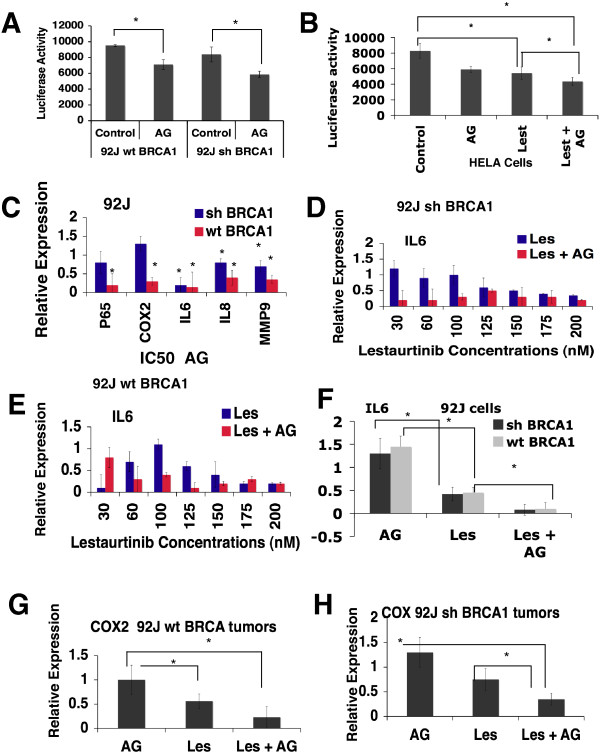
**AG14361 inhibits NF-κB activity and downregulates the levels of expression of NF-κB downstream genes. (A)** Luciferase reporter assay for NF-κB transcription activity after treatment with IC_50_ of AG14361 for 24 hr in 92 J cell lines. **(B)** NF-κB transcription activity assay after treatment with IC_50_ of AG14361 and/or lestaurtinib for 24 hr in a HeLa cell line, which carries a stably integrated luciferase reporter. **(C)** The real-time PCR of five NF-κB downstream genes in 92 J cells. **(D, E)** Expression of *IL6* in 92 J cells in the presence of AG14361 and various concentrations of lestaurtinib for 24 hr revealed by real-time PCR. **(F-H)** Expression of *IL6* and *COX2* in xenograft tumors after treatment of AG14361 and/or lestaurtinib. Data shown the mean of three different readings and are marked by columns with ± standard deviation (SD) error bars. IC_50_, 50% inhibition dose; NF-κB, nuclear factor κB.

We previously identified some compounds in this library that inhibit NF-κB signaling [47]. Among the eleven compounds confirmed in our secondary screen that exhibit strong synergy with PARPi in killing breast cancers irrespective of their BRCA1 status, four can inhibit NF-κB activity (lestaurtinib, bortezomib, ouabain, and digitoxin). These data suggest that inhibition of NF-κB might be one of the reasons for the synergistic killing of these cancer cells with PARPi. To investigate this theory, we analyzed the effect of AG14361 on NF-κB transcriptional activity using a luciferase reporter. The data showed that AG14361 treatment inhibited transcriptional activity of both shBRCA1 and wtBRCA1 cells (Figure 6A). Next, we investigate the effect of AG14361 on NF-κB signaling by investigating the expression levels of several downstream genes. We found that treatment of AG14361 at its IC_50_ significantly repressed expression of all the genes tested in wtBRCA1 cells; whereas in shBRCA1 cells, AG14361 could significantly repress expression of *IL-6*, *IL8*, and *MMP9*, but did not significant change expression of *p65* and *COX2* (Figure 6B). These data show that AG14361 at its IC_50_ concentration could affect expression of some transcriptional downstream genes of NF-κB although a stronger effect was observed in wtBRCA1 cells than in shBRCA1 cells.

Next we investigated whether the inhibitory effect of NF-κB by AG14361 was potentiated by the presence of lestaurtinib by examining the expression of a NF-κB luciferase reporter that is stably integrated into the genome of HeLa cells. The data indicated that while mono-treatment of AG14361 and lestaurtinib at their IC_50_ significantly inhibited the NF-κB transcriptional activity, the combination of both enhanced the effect (Figure 6C). We then examined the expression of several downstream genes, *IL-6*, *IL8*, *p50*, and *COX-2*. Our data indicated that lestaurtinib inhibited expression of these genes in a dose-dependent manner and this effect was significantly enhanced in the presence of AG14361 at its IC_50_ concentration (Figure 6D,E, and Additional file 4). We have also examined the expression of these genes in the tumors treated with AG14361 and/or lestaurtinib that were harvested at their end point. The data indicated that AG14361 mono-treatment had no obvious effect on *IL-6* and *COX2* expression, whereas lestaurtinib significantly inhibited their expression. This effect was enhanced with the combined treatment with both drugs (Figure 6F-I).

## Discussion

The majority of BRCA1/2-related breast cancers exhibit high grade and are insensitive to most available hormonal or targeted therapeutic agents [1-7,48,49]. Moreover many sporadic breast cancers also exhibit reduced or diminished expression of BRCA1 [50,51]. Thus the finding that BRCA1/2-associated breast cancers are highly sensitive to PARPi has been considered as a very promising approach for breast cancer [20-24,52]. However, like many other therapeutic agents, PARPi treatment is also associated with drug resistance after initial response to the treatment (reviewed in [31]). For example, in phase II trials, 400 mg twice daily exposure of PARPi olaparib only resulted in the delay of breast cancer and ovarian cancer progression (both with median progression-free survival of about six months), and all patients with BRCA1/2 mutations eventually died of cancers [53,54].

In mouse models, prolonged treatment of the PARPi, AZD2281 also resulted in drug resistance presumably due to upregulation of Abcb1a/b genes encoding P-glycoprotein efflux pumps [36]. Do Soto *et al*. [28] found that while a PARPi was able to kill naïve BRCA1 mutant cells with high specificity both *in vitro* and *in vivo*, it exhibited minimal specificity in inhibiting several cell lines derived from mouse Brca1 mutant mammary tumors. Altogether, these observations reinforced the need for screens for additional drugs that efficiently kill BRCA1/2-associated cancer cells when combined with PARPi. Of note, it has been demonstrated that PARPi, when combined with agents that impair DNA repair, are also effective in killing cancer cells containing wild-type BRCA1/2 [36,55,56].

In this study, we screened a library that contains 2,816 small molecules, most of which are approved for human or animal use by the FDA or other countries [39,47], in the presence of AG14361 at a constant sublethal dose in order to identify compounds that kill breast cancer cells synergistically with PARPi. Our initial screen identified seventeen compounds that have similar levels in killing both shBRCA1 and wtBRCA1 cells, six compounds that are more specific for killing shBRCA1 cells, and nine compounds that kill wtBRCA1 cells better. After validation, lestaurtinib was selected for further investigation. Lestaurtinib is an orally bioavailable multikinase inhibitor for a number of kinases including protein kinase C-related kinase 1 (PRK1) [57], FMS-like tyrosine kinase 3 (FLT3) [44,45], JAK2 [46,58], Trk-A/B/C [59,60]. Lestaurtinib has been used in clinical trials for myeloproliferative disorders, and acute myelogenous leukemia [44-46], but there has been no report of its application for breast cancer treatment yet. Our data indicated that lestaurtinib is highly potent against tumor cells derived from both mouse and human breast cancers as a mono-treatment agent. In combination with AG14361, this effect is synergistically enhanced as reflected by further delay of tumor progression. We also found that four out of fourteen tumors completely regressed during combination treatment, while no regression was observed in the other three groups of mice (control, mono-treatment of either AG14361 or lestaurtinib) carrying a total of 42 tumors derived from each of cell line tested. Therefore, the synergy between AG14361 and lestaurtinib treatment is significant in these cancer cells. The complete tumor regression in these four groups of animals may reflect a differential threshold response of these mice to the treatment compared with the other recipients. In the clinic setting, complete tumor regression upon the therapeutic treatment is the most desirable outcome, however it does not always happen. In most cases, patients display partial response at different degrees, perhaps, due to individual difference in response to the treatment [53,54]. Nonetheless, the significant delay of tumor progression could prolong the life of patients and provide valuable time for further therapeutic therapies. Our data are reminiscent of this feature. We are in the process of screening further drug combinations in order to achieve the most desirable outcome in the near future.

The effect of lestaurtinib are primarily on G2 arrest, apoptosis and reduced proliferation of cancer cells irrespective of their BRCA1 status. Mono-treatment of AG14361 exhibited a similar, yet mild effect on apoptosis and proliferation; however, it affected all phases of the cell cycle. Of note, the combination of both drugs results in dramatic expansion of cells in the G2 phase at the expense of the S phase. This may account for the much more severe growth retardation and markedly enhanced cell death. Of note, we found that four out of eleven compounds, including lestaurtinib, which exhibits synergy with PARPi, could inhibit NF-κB activity based on our previous study [47]. NF-κB is a transcription factor that plays important roles in cell cycle progression, cell survival and inflammation [1,52,59,60]. Therefore we tested the effect of AG14361 on NF-κB and found it could also inhibit NF-κB activity, albeit to a less extent compared with lestaurtinib. When combined together, AG14361 and lestaurtinib exhibited a much stronger inhibitory effect on the expression of a number of genes in the NF-κB signaling pathway, such as *p50*, *p65*, *IL6*, *IL8*, *COX2* and *MMP9* that are involved in cancer cell proliferation, inflammation, invasion and/or cell death [45,50,54,58,59].

## Conclusions

Our data indicated that lestaurtinib is a potent therapeutic agent for killing breast cancer cells and it amplifies the ability of the PARP1 inhibitor AG14361 to kill breast cancer cells irrespective to their BRCA1 status. This effect is, at least in part, by inhibiting NF-κB signaling. Because lestaurtinib and PARPi are drugs approved for clinical trials for several different cancers [44-46,53,54], we believe this combination will be applicable for a breast cancer trial in the near future.

## Abbreviations

BRCA1/2: breast cancer type 1/2 susceptibility; DMEM: Dulbecco’s modified Eagle’s medium; DSBs: double-strand breaks; HR: homologous recombination; IC_50_: 50% inhibition dose; NF-κB: nuclear factor κB; PARP1: poly(ADP-ribose)polymerase 1; PARPi: PARP inhibitor; PBS: phosphate-buffered saline; PCNA: proliferating cell nuclear antigen; qRT-PCR: quantitative reverse transcription-polymerase chain reaction; shBRCA1: sample with short hairpin RNA (shRNA) against BRCA1; SSBs: single-strand breaks; TUNEL: terminal deoxynucleotidyl transferase-mediated dUTP-biotin nick end-labeling; wtBRCA1: sample with wild-type BRCA1 status.

## Competing interests

The authors declare that they have no competing interests.

## Authors’ contributions

GVO designed and performed the experiments, analyzed and interpreted the data and wrote the manuscript. CC, SS and TJL performed the experiments. XX developed the cell lines. CL performed the mice surgeries, CJT synthesized the AG14361. RH and MHX designed the high-throughput experiments and performed the data analysis. CXD designed the experiments, interpreted the data and wrote the manuscript. CJT, MHX and CXD revised the manuscript for important intellectual content. All authors read and approved the final manuscript.

## Supplementary Material

Additional file 1 **Murine cell lines Ras and 69 proliferation *in vitro* and tumor growth in allografts. (A)** Fold change in the cell growth rate in respect to the initial inoculated Ras and 69 cells lines. Data shown represent mean standard deviation (SD) from triplicate experiments. **(B)** Example of clonogenic assay performed on Ras and 69 cell lines. A total of 1,000 cells were seeded in 10 ml plates and grown for 21 days and counted at the end point. Data represent the mean SD from triplicate experiments. **(C)** Cells were injected into the mammary fat pad of nude mice and the tumor volume was measured until they reached 3.5 mm^3^. Each group consisted of five mice and each mouse had two tumors, the measurements on the graph represent the average of ten tumors per group. Click here for file

Additional file 2: Table S1 Identification of clinically used drugs that kill 92 J isogenic pair as single agents and show the synergistic toxic effect with AG14361. Click here for file

Additional file 3 **Cell viability assay of deserpiline that was eliminated for further analysis after the cherry pick. (A)** Viability assay of deserpiline from the primary high throughput screen. **(B)** Viability assay of the same drug from the secondary screen. The synergy is less obvious in the secondary screen as some points of the curve touch, or even cross with the expected additive values curve. Click here for file

Additional file 4 **Synergistic effect of lestaurtinib in combination with AG14361 in Ras and 69 cell lines.** Expression of *COX2***(A,B)**, *IL8***(C,D)**, and *p50***(E,F)** in 92 J pair of isogenic cells in the presence of AG14361 and/or various concentrations of lestaurnitib for 24 hr revealed by real-time PCR. Data show the mean of three different readings and are marked by columns with ± standard deviation (SD) error bars. Click here for file
