# Supplementary material for: Drug repurposing screen identifies lestaurtinib amplifies the ability of the poly (ADP-ribose) polymerase 1 inhibitor AG14361 to kill breast cancer associated gene-1 mutant and wild type breast cancer cells
Source: Breast Cancer Res. 2014 Jun 24;16(3):R67. doi: 10.1186/bcr3682 (PMC4229979; doi:10.1186/bcr3682)
Supplement: Additional file 1 — Murine cell lines Ras and 69 proliferation in vitro and tumor growth in allografts. (A) Fold change in the cell growth rate in respect to the initial inoculated Ras and 69 cells lines. Data shown represent mean standard deviation (SD) from triplicate experiments. (B) Example of clonogenic assay performed on Ras and 69 cell lines. A total of 1,000 cells were seeded in 10 ml plates and grown for 21 days and counted at the end point. Data represent the mean SD from triplicate experiments. (C) Cells were injected into the mammary fat pad of nude mice and the tumor volume was measured until they reached 3.5 mm3. Each group consisted of five mice and each mouse had two tumors, the measurements on the graph represent the average of ten tumors per group. [file bcr3682-S1.pdf]

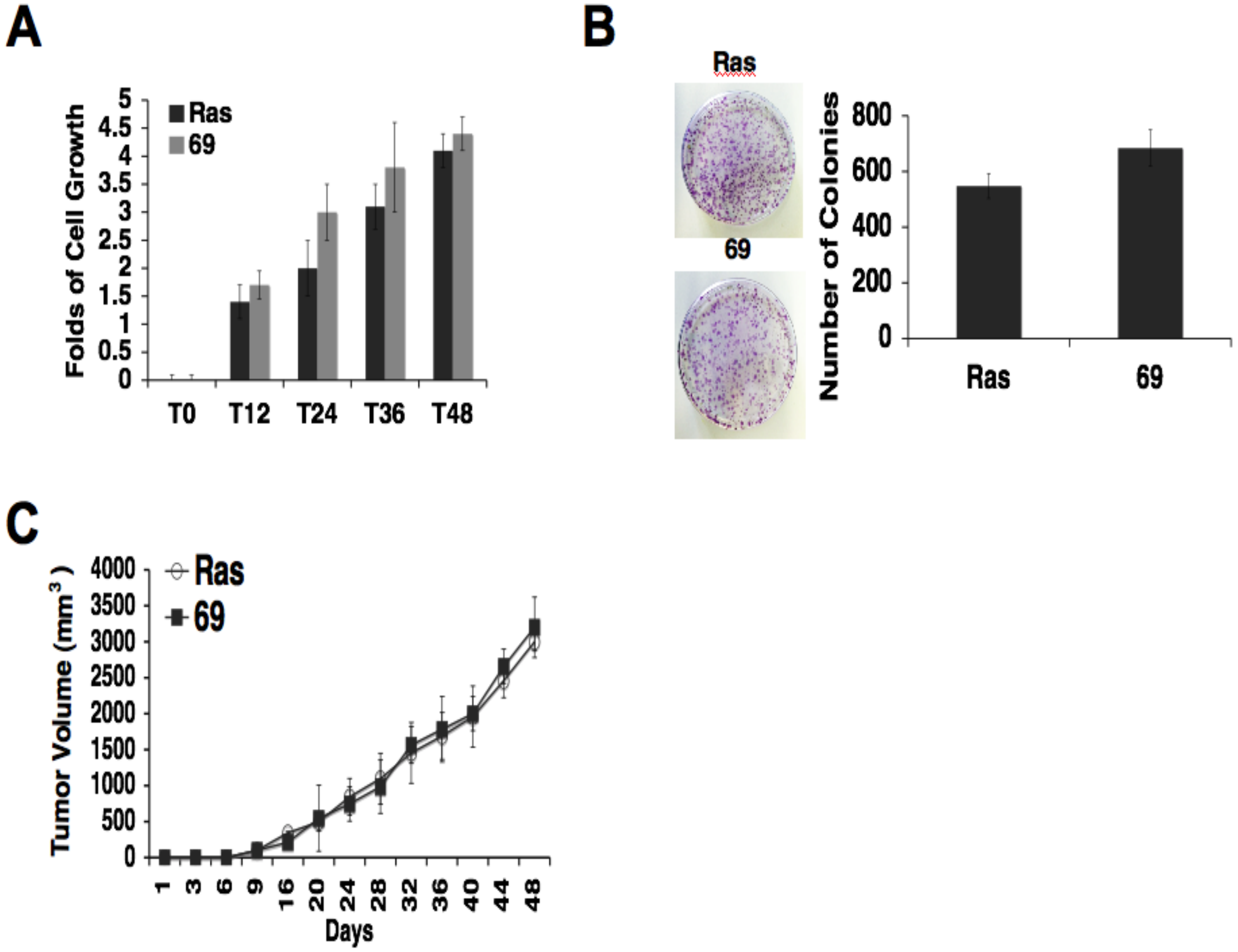

Additional File 1. Murine cell lines Ras and 69 proliferation in vitro and tumor growth in allografts. **(A)** Fold change in the cell growth rate in respect to the initial inoculated Ras and 69 cells lines. Data shown represent mean SD from triplicate experiments. **(B)** Example of clonogenic assay performed on Ras and 69 cell lines. A total of 1000 cells were seeded in 10 ml plates and grown for 21 days and counted at the end point. Data represent the mean SD form triplicate experiments. **(C)** Cells were injected into the mammary fat pad of nude mice and the tumor volume was measured until they reached 3.5 mm^3. Each group consisted of five mice and each mouse had two tumors, the measurements on the graph represent the average of ten tumors per group.
