# Supplementary material for: Drug repurposing screen identifies lestaurtinib amplifies the ability of the poly (ADP-ribose) polymerase 1 inhibitor AG14361 to kill breast cancer associated gene-1 mutant and wild type breast cancer cells
Source: Breast Cancer Res. 2014 Jun 24;16(3):R67. doi: 10.1186/bcr3682 (PMC4229979; doi:10.1186/bcr3682)
Supplement: Additional file 2: Table S1 — Identification of clinically used drugs that kill 92 J isogenic pair as single agents and show the synergistic toxic effect with AG14361. [file bcr3682-S2.docx]

**Additional file 2: Table S1. Identification of clinically used drugs that kill 92J isogenic pair as single agents and also show synergistic toxic effect with AG14361.**

| **Drug name** | **Identificator** | **Combination Index (Synergy)** | |
| --- | --- | --- | --- |
|  |  | **92J- sh-BRCA1** | **92J-wt BRCA1** |
| Lestaurtinib | NCGC00168772-01 | +++++ | ++++ |
| Bortezomib | NCGC00181022-01 | ++++ | +++++ |
| Orlistat | NCGC00095128-01 | +++++ | ++++ |
| TOMATINE | NCGC00095843-01 | ++++ | ++++ |
| PIMOZIDE | NCGC00093976-03 | ++++ | ++++ |
| PROSCILLARIDIN A | NCGC00016447-01 | +++ | +++ |
| OUABAIN | NCGC00163473-01 | +++ | +++ |
| DIGITOXIN | NCGC00159428-02 | +++ | +++ |
| METILDIGOXIN | NCGC00168746-01 | +++ | +++ |
| LANATOSIDE A | NCGC00160523-01 | +++ | +++ |
| LANATOSIDE C | NCGC00094932-01 | +++ | +++ |
| DESERPIDINE | NCGC00168786-01 | +++ | +++ |
| AMLEXANOX | NCGC00167472-01 | +++ | +++ |
| ETHOXAZENE HYDROCHOLIDE | NCGC00160548-01 | +++ | +++ |
| DESLANOZIDE | NCGC00159441-02 | +++ | +++ |
| ZINC PYRITHIONE | NCGC00091933-01 | +++ | +++ |
| POTASSIUM DICHROMATE | NCGC00090755-02 | +++ | +++ |
| THONZONIUM BROMIDE | NCGC00016507-01 | ++ | ++++ |
| THIONOSIDE | NCGC00096122-03 | ++ | +++ |
| 6-MERCAPTOPURINE MONOHYDRATE | NCGC00091641-01 | + | +++ |
| SB 206553 hYDROCHLORIDE | NCGC00094422-01 | + | +++ |
| CETYLTRIMETHYLAMONIUM BROMIDE | NCGC00164283-01 | + | +++ |
| BENZETHONIUM BROMIDE | NCGC00091528-02 | + | +++ |
| ARIPIPRAZOLE | NCGC00159510-02 | + | +++ |
| CLOFOCTOL | NCGC00095016-01 | + | +++ |
| VANDETANIB | NCGC00167513-01 | + | +++ |
| Cantharidin | NCGC00016247-01 | ++++ | + |
| Chloroquine diphosphate | NCGC00015256-01 | ++++ | + |
| Amodiaquin dihydrochloride dihydrate | NCGC00017063-01 | ++++ | + |
| CERIVASTATIN SODIUM | NCGC00164625-02 | +++ | ++ |
| Pitavastatin | NCGC00164566-01 | +++ | ++ |
| Fluvastatina | NCGC00164604-01 | +++ | + |

| **Range of combination index** | **description** | **grad simbols** |
| --- | --- | --- |
| **<0.1** | very strong synergism | +++++ |
| **0.1 – 0.3** | strong synergism | ++++ |
| **0.3 – 0.7** | synergism | +++ |
| **0.85 – 0.90** | slight synergism | ++ |
| **0.90 – 1.10** | nearly additive | + |
| **1.10 – 1.20** | slight antagonism | - |
