# Supplementary material for: Drug repurposing screen identifies lestaurtinib amplifies the ability of the poly (ADP-ribose) polymerase 1 inhibitor AG14361 to kill breast cancer associated gene-1 mutant and wild type breast cancer cells
Source: Breast Cancer Res. 2014 Jun 24;16(3):R67. doi: 10.1186/bcr3682 (PMC4229979; doi:10.1186/bcr3682)
Supplement: Additional file 3 — Cell viability assay of deserpiline that was eliminated for further analysis after the cherry pick. (A) Viability assay of deserpiline from the primary high throughput screen. (B) Viability assay of the same drug from the secondary screen. The synergy is less obvious in the secondary screen as some points of the curve touch, or even cross with the expected additive values curve. [file bcr3682-S3.pdf]

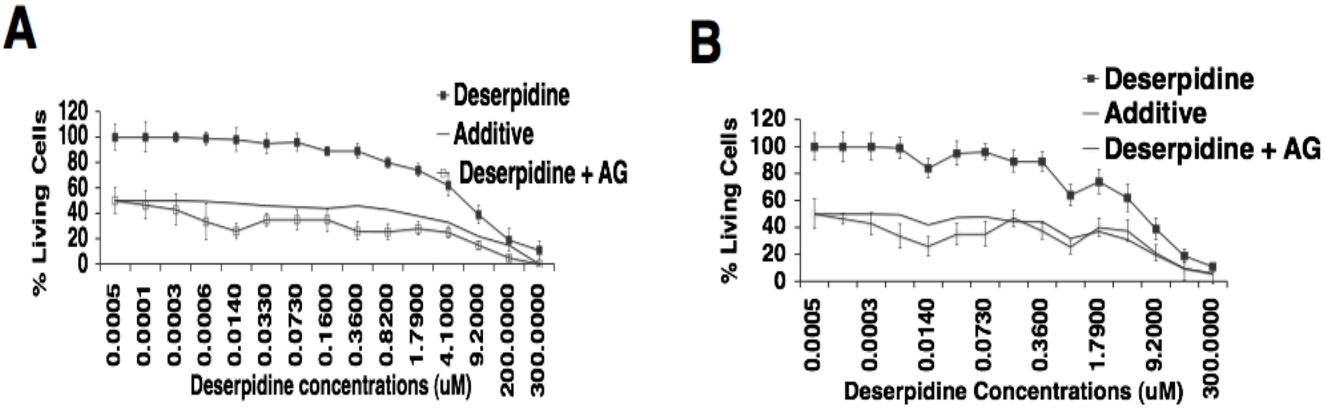

Additional File 3. Cell viability assay of deserpiline that was eliminated for further analysis after the cherry pick. (A) Viability assay of deserpiline from the primary high throughput screen. **(B)** viability assay of the same drug from the secondary screen. The synergy is less obvious in the secondary screen as some points of the curve touch, or even cross with the expected additive values curve.
