# Supplementary material for: Drug repurposing screen identifies lestaurtinib amplifies the ability of the poly (ADP-ribose) polymerase 1 inhibitor AG14361 to kill breast cancer associated gene-1 mutant and wild type breast cancer cells
Source: Breast Cancer Res. 2014 Jun 24;16(3):R67. doi: 10.1186/bcr3682 (PMC4229979; doi:10.1186/bcr3682)
Supplement: Additional file 4 — Synergistic effect of lestaurtinib in combination with AG14361 in Ras and 69 cell lines. Expression of COX2(A,B), IL8(C,D), and p50(E,F) in 92 J pair of isogenic cells in the presence of AG14361 and/or various concentrations of lestaurnitib for 24 hr revealed by real-time PCR. Data show the mean of three different readings and are marked by columns with ± standard deviation (SD) error bars. [file bcr3682-S4.pdf]

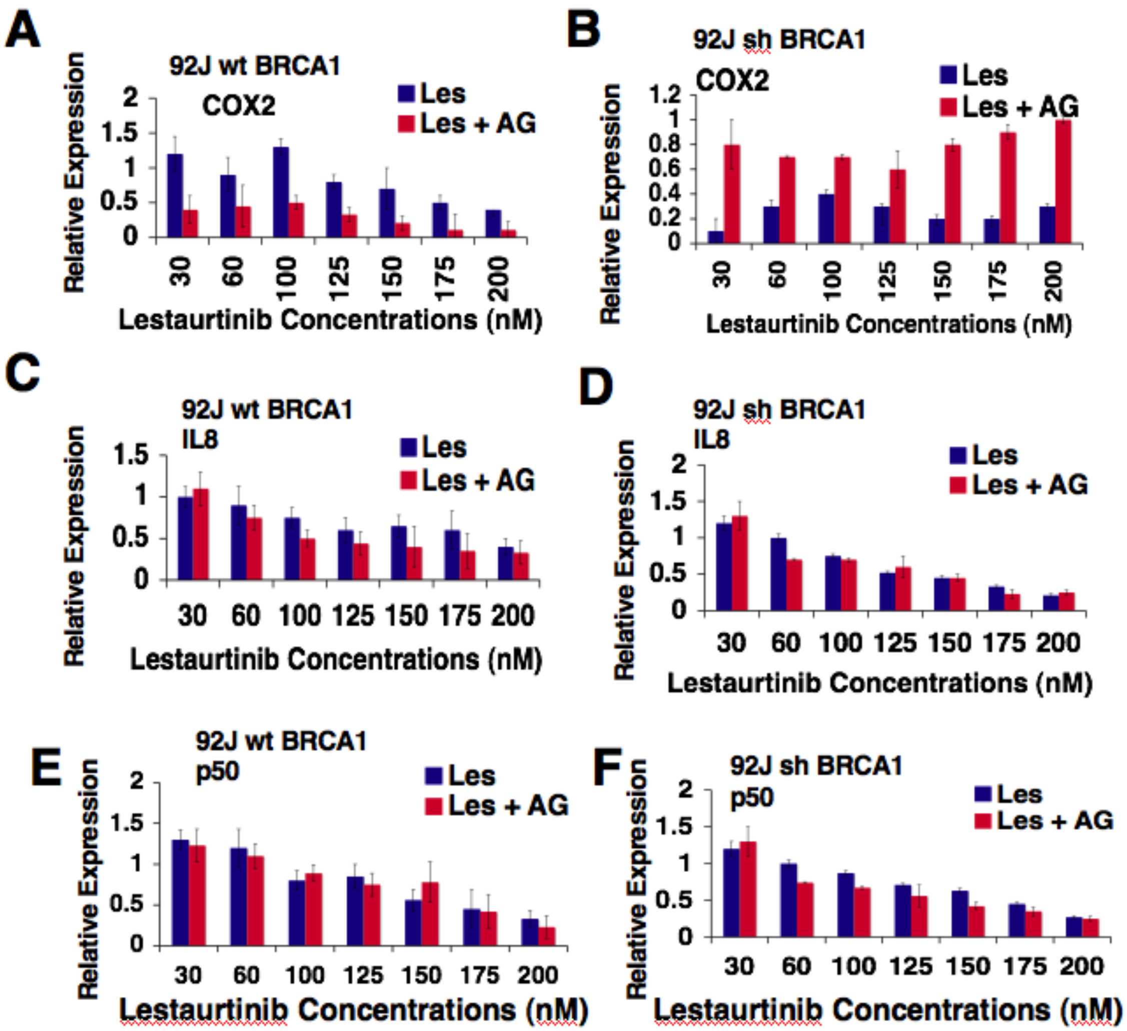

Additional File 4. Synergistic effect of lestaurtinib in combination with AG14361 in Ras and 69 cell lines. Expression of *COX2* **(A,B),** *IL8* **(C,D),** and *p50* **(E,F)** in 92J pair of isogenic cells in the presence of AG14361 and/or various concentrations of lestaurnitib for 24 hr revealed by real-time PCR. Data show the mean of three different readings and are marked by columns with ± SD error bars.
